# Epigenetic regulation in cancer chemoresistance and combined therapeutic strategies

**DOI:** 10.3389/fcell.2026.1814084

**Published:** 2026-04-13

**Authors:** Lili Jiang, Yixiao Yuan, Dahang Zhang, Juan Wang, Xiulin Jiang, Yuqiang Shi

**Affiliations:** 1 Department of Urology, Aerospace Center Hospital, Beijing, China; 2 The Third Affiliated Hospital of Kunming Medical University, Peking University Cancer Hospital Yunnan, Kunming, Yunan, China; 3 College of Life Science, University of Chinese Academy of Sciences, Beijing, China

**Keywords:** cancer stem cells, chemoresistance, combination therapy, DNA methylation, epigenetics, histone modification, inhibitors, non-coding RNA

## Abstract

Chemotherapy remains a cornerstone treatment for various malignancies; however, its efficacy is often limited by the development of drug resistance, which increases the risk of tumor recurrence and metastasis. Chemoresistance arises from multiple mechanisms, including enhanced drug efflux, apoptosis inhibition, increased DNA damage repair, and the maintenance of cancer stem cells (CSCs). Recent studies have revealed that epigenetic alterations play a critical role in chemoresistance. DNA methylation, histone modifications, and non-coding RNAs contribute to resistance by regulating gene expression, signaling pathways, and CSC properties. RNA epigenetic modifications, such as N6-methyladenosine (m^6^A), N4-acetylcytidine (ac^4^C), and 5-methylcytidine (m^5^C), regulate mRNA stability, splicing, and translation efficiency, thereby sustaining CSC self-renewal and promoting resistance. Epigenetic-targeted agents, including DNA methyltransferase inhibitors (DNMTi), histone deacetylase inhibitors (HDACi), and emerging inhibitors targeting RNA-modifying enzymes, have demonstrated the potential to reverse resistance, suppress CSC traits, and enhance chemosensitivity *in vitro* and *in vivo*. Combining epigenetic drugs with conventional chemotherapy enables multi-level intervention in resistance mechanisms, significantly improving therapeutic outcomes and offering new avenues for personalized cancer treatment. Future studies should focus on developing precise biomarkers, optimizing combination strategies, and conducting clinical validation to advance the application of epigenetic interventions in chemoresistant cancers.

## 1 Introduction

Chemotherapy is a cornerstone treatment for many malignancies, but its efficacy is often limited by the development of drug resistance (Kris et al., 2014). Chemoresistance not only leads to initial treatment failure but also significantly increases the risk of tumor recurrence and metastasis, thereby severely affecting overall survival and prognosis (Yeldag et al., 2018). Resistance arises from multiple, layered mechanisms, including enhanced drug efflux, inhibition of apoptosis, increased DNA damage repair, and the maintenance of CSC populations (Yeldag et al., 2018). Traditional studies have largely focused on resistance driven by genetic mutations. However, accumulating evidence in recent years indicates that epigenetic alterations also play a critical role in chemotherapy resistance (Gu et al., 2025; Chen et al., 2024). Unlike irreversible genetic mutations, epigenetic modifications—such as DNA methylation, histone modifications, and non-coding RNA regulation—are reversible and dynamically tunable (Mosca et al., 2021; Zhao et al., 2020). This property provides unique opportunities for therapeutic intervention. For instance, modulating specific epigenetic marks can restore drug sensitivity or suppress resistance-associated signaling pathways, thereby improving treatment outcomes (Sawant et al., 2023). Moreover, epigenetic mechanisms can dynamically respond to external drug pressure, allowing tumor cells to rapidly adapt to the chemotherapeutic environment, making them a key factor in resistance development (Mosca et al., 2021; Sritharan and Sivalingam, 2025).

This Mini Review summarizes the roles of epigenetic alterations in cancer chemoresistance and highlights recent advances in epigenetic-targeted therapies. Compared with previous reviews, this work provides an updated perspective by incorporating RNA epigenetic modifications into the conventional epigenetic framework. In particular, emerging epitranscriptomic regulations, including m^6^A, m^5^C, and ac^4^C, are discussed alongside classical mechanisms such as DNA methylation and histone modifications, thereby expanding the scope of epigenetic regulation in chemoresistance. In addition, this review emphasizes the therapeutic potential of targeting RNA epigenetic regulators in combination with conventional chemotherapeutic agents, suggesting that such strategies may offer improved efficacy in overcoming drug resistance. We further outline the underlying rationale as well as emerging preclinical and clinical evidence supporting these combination approaches. By integrating these aspects, this review provides a broader and more forward-looking perspective on epigenetic regulation and offers insights into the development of more effective combination therapies for drug-resistant cancers.

## 2 Mechanisms by which epigenetic alterations drive chemoresistance in cancer cells

**FIGURE 1:**
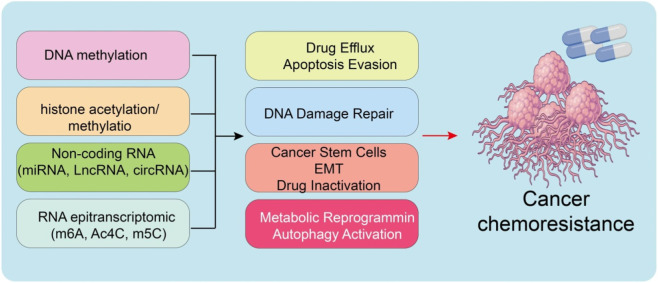
Overview of combining epigenetic regulator inhibitors with synergistic chemotherapeutic agents to inhibit cancer progression. Epigenetic inhibitors, including DNMT inhibitors (DNMTi) targeting DNA methylation, HDAC inhibitors (HDACi) targeting histone acetylation, antisense oligonucleotides or siRNAs (ASO/siRNA) targeting lncRNAs/circRNAs, and RNA modification inhibitors (m^6^A i, m^5^C i, ac^4^C i) targeting m^6^A, m^5^C, and ac^4^C modifications, can enhance the efficacy of conventional chemotherapeutics. This combination suppresses multiple cancer-promoting processes such as drug efflux, apoptosis evasion, DNA damage repair, cancer stem cell maintenance, EMT, drug inactivation, metabolic reprogramming, and autophagy activation, ultimately leading to inhibition of cancer progression.

In Figure 1, we illustrates a conceptual framework for combining epigenetic regulator inhibitors with synergistic chemotherapeutic agents to enhance anticancer efficacy. The schematic highlights how targeting DNA methylation (DNMTi), histone acetylation (HDACi), noncoding RNAs (ASO/siRNA), and RNA modifications (m^6^A, m^5^C, ac^4^C inhibitors) can potentiate conventional chemotherapy. By simultaneously disrupting multiple cancer-promoting processes, including drug efflux, apoptosis resistance, DNA repair, CSC maintenance, EMT, drug inactivation, metabolic reprogramming, and autophagy, this combinatorial strategy effectively suppresses cancer progression.

### 2.1 DNA methylation

DNA methylation is one of the earliest discovered forms of epigenetic modification (Moore et al., 2013). It predominantly occurs at cytosine-phosphate-guanine (CpG) islands and is catalyzed by DNMTs, which add methyl groups to cytosine residues to regulate gene expression (Moore et al., 2013). Aberrant DNA methylation in cancer cells is a major mechanism driving chemoresistance. On one hand, hypermethylation can silence key drug targets or pro-apoptotic genes (Moore et al., 2013). For example, hypermethylation of promoters of drug-dependent receptors or transporters reduces their expression, impairing the efficacy of chemotherapeutic agents (Moore et al., 2013). On the other hand, hypomethylation or demethylation can activate resistance-related genes, such as those encoding drug efflux pumps (e.g., the ABC protein family) or DNA damage repair enzymes, thereby enhancing the tumor cell’s ability to eliminate drugs and repair DNA (Kulis and Esteller, 2010). Importantly, DNA methylation also plays a crucial role in the maintenance and plasticity of CSCs. Aberrant methylation patterns can regulate the expression of stemness-associated genes (such as OCT4, SOX2, and NANOG), thereby sustaining CSC self-renewal and tumor-initiating capacity (Fang X. et al., 2025). In addition, dynamic changes in DNA methylation enable non-CSCs to acquire stem-like properties under therapeutic pressure, contributing to tumor relapse and resistance (Al-Imam et al., 2023). Furthermore, the dynamic plasticity of DNA methylation allows cancer cells to rapidly adjust their gene expression profiles under chemotherapeutic pressure, facilitating adaptation to the drug environment (Kulis and Esteller, 2010). This feature not only promotes the development of resistance but also offers potential opportunities for clinical intervention. For instance, DNMTi such as Azacitidine and Decitabine have been applied in certain hematologic malignancies to reactivate silenced tumor suppressor genes through demethylation, thereby restoring drug sensitivity (Hu et al., 2021). Notably, these agents may also impair CSC maintenance by reactivating differentiation-related pathways, suggesting a dual role in targeting both bulk tumor cells and CSC populations (Wainwright and Scaffidi, 2017). Future studies are needed to elucidate the specific targets and mechanisms of DNA methylation in different cancer types and treatment contexts to guide personalized epigenetic intervention strategies.

### 2.2 Histone modifications

Histone modifications represent a central layer of epigenetic regulation, encompassing a variety of reversible chemical changes such as acetylation, methylation, phosphorylation, and ubiquitination, which collectively shape chromatin structure and transcriptional activity (Zaib et al., 2022). Histone acetylation, typically mediated by histone acetyltransferases (HATs), is generally associated with transcriptional activation through chromatin relaxation, whereas histone deacetylation by histone deacetylases (HDACs) promotes chromatin compaction and gene repression (Shvedunova and Akhtar, 2022; Narita et al., 2019). Histone methylation exerts context-dependent effects depending on the specific residues and methylation states (Bilme et al., 2022). For example, H3K4me3 and H3K36me3 are commonly linked to active transcription, while H3K27me3 and H3K9me3 are associated with transcriptional repression and heterochromatin formation{Xiong, 2024 #1220; (Ji et al., 2025). In addition, other modifications such as histone phosphorylation and ubiquitination contribute to DNA damage response, chromatin remodeling, and transcriptional fine-tuning (Armache et al., 2020; Mattiroli and Penengo, 2021). Histone lactylation is a recently identified epigenetic modification derived from lactate, linking cellular metabolism to chromatin regulation (L et al., 2024). It can directly activate gene transcription and has been implicated in tumor progression and chemoresistance by promoting adaptive gene expression programs (L et al., 2024). Given the lactate-rich tumor microenvironment, histone lactylation may also contribute to the maintenance of cancer stem cell properties and cellular plasticity.

These alterations frequently result in the activation of anti-apoptotic pathways, enhancement of DNA repair capacity, and upregulation of drug resistance-associated genes (Christmann and Kaina, 2019). Importantly, histone modifications also play a critical role in regulating CSC properties, as specific chromatin states can maintain stemness-related gene expression programs and enable cellular plasticity under therapeutic pressure (Jin and Jeong, 2023). The reversibility of histone modifications makes them attractive therapeutic targets. Inhibitors targeting HDACs, histone methyltransferases (e.g., EZH2), and demethylases have shown the ability to remodel chromatin states, restore drug sensitivity, and suppress tumor growth in preclinical and clinical settings (Fontecha-Barriuso et al., 2018). Overall, targeting histone modifications represents a promising strategy to overcome chemoresistance through coordinated regulation of tumor cell survival, epigenetic plasticity, and stemness-associated programs. Importantly, histone modifications are also key regulators of CSC identity and function (Milan et al., 2022). Epigenetic enzymes such as histone methyltransferases and deacetylases can modulate chromatin states at promoters or enhancers of stemness-associated genes, thereby maintaining CSC self-renewal, pluripotency, and tumor-initiating potential (Luo et al., 2024; Zhou et al., 2024). For instance, specific histone modification patterns (e.g., H3K27ac or H3K4me3 enrichment) are frequently associated with active transcription of CSC-related gene programs, while repressive marks can dynamically regulate CSC differentiation (Wang Y. et al., 2025). Moreover, the reversibility of histone modifications provides opportunities for clinical intervention. HDACi can restore drug sensitivity by remodeling chromatin and suppressing expression of resistance-associated genes (Zhao and Zhang, 2019). Notably, these inhibitors may also disrupt CSC maintenance by altering the epigenetic landscape that supports stemness. For instance, nuclear pyruvate dehydrogenase complex (nPDC) activity is persistently inhibited by the nuclear protein ELMSAN1 (Zhao et al., 2025). Pharmacological targeting of the ELMSAN1–nPDC interaction can enhance nPDC activity, increase nuclear acetyl-CoA levels, promote histone acetylation, drive cells into post-mitotic states, and reduce primitive cell characteristics (Zhao et al., 2025). Combined inhibition of HDAC1/2 further reinforces this epigenetic reprogramming, significantly suppressing tumor growth, reducing tumor-initiating capacity, and improving survival in multiple drug-resistant cancer models, indicating that modulation of nuclear acetyl-CoA and histone acetylation can effectively overcome chemotherapy resistance (Zhao et al., 2025).

### 2.3 Non-coding RNAs (miRNA, lncRNA, circRNA)

Non-coding RNAs (ncRNAs), including microRNAs (miRNAs), long non-coding RNAs (lncRNAs), and circular RNAs (circRNAs), play critical regulatory roles in chemoresistance (Hombach and Kretz, 2016). miRNAs can modulate drug sensitivity by targeting mRNAs of resistance-related genes, promoting their degradation or inhibiting translation (Hombach and Kretz, 2016). lncRNAs and circRNAs can act as miRNA sponges, regulate chromatin structure, or interact with transcription factors to influence drug tolerance (Hombach and Kretz, 2016). Furthermore, ncRNAs can regulate CSC maintenance and function, supporting survival and recurrence of drug-resistant cells. For example, certain lncRNAs have been shown to activate Wnt/β-catenin, Notch, or Hedgehog signaling pathways, maintaining CSC phenotypes and enhancing chemoresistance (Gao et al., 2023). Our previous study demonstrated that LncRNA-PKMYT1AR is significantly upregulated in NSCLC, acting as a sponge for miR-485-5p, thereby increasing PKMYT1 expression, stabilizing β-catenin, activating Wnt/β-catenin signaling, and promoting cisplatin resistance (He et al., 2021). Additionally, we recently identified circRNA-circ0515, which serves as an RNA–protein scaffold, facilitating RBM45 binding to SDHB, enhancing SDHB stability, and regulating mitochondrial succinate metabolism, thereby promoting cisplatin resistance in lung cancer (Yuan et al., 2025). Collectively, ncRNAs represent key nodes in chemoresistance mechanisms and potential molecular targets for predictive and therapeutic interventions.

### 2.4 RNA epigenetic modifications and cancer stem cells

Beyond DNA methylation and histone modifications, RNA epigenetic modifications (epitranscriptomics) play critical roles in CSC maintenance and chemoresistance (Zhao et al., 2020). RNA modifications include N^6^-methyladenosine, 5-hydroxymethylcytidine, and N^4^-acetylcytidine, which fine-tune gene expression by regulating RNA stability, splicing, translation efficiency, and subcellular localization (Delaunay et al., 2024). Among these, m^6^A is the most extensively studied. It is “written” by methyltransferase complexes such as METTL3/METTL14, “erased” by demethylases such as FTO and ALKBH5, and “read” by specific binding proteins such as the YTHDF family (Jia et al., 2021). In CSCs, m^6^A modification regulates the stability and translation of key stemness genes, including SOX2 and MYC, thereby sustaining CSC self-renewal and multipotency (Wang A. et al., 2023). For instance, YTHDF1 promotes translation of m^6^A-modified NOTCH1, driving liver cancer stem cell traits and resistance to tyrosine kinase inhibitors, representing a potential therapeutic target (Zhang X. et al., 2024). YTHDF2 stabilizes m^6^A -modified MYC and VEGFA mRNAs in glioblastoma stem cells, promoting stemness and tumor growth via downstream IGFBP3, offering a glioblastoma-specific therapeutic strategy (Dixit et al., 2021). Dysregulated m^6^A modification enhances CSC tolerance to chemotherapeutics and promotes tumor recurrence and metastasis. Other RNA modifications, including ac^**4**^C and hm^5^C, also modulate CSC-related signaling pathways, such as Wnt/β-catenin, Notch, and Hedgehog, affecting CSC survival and proliferation (Cerneckis et al., 2023). Targeting RNA-modifying enzymes, such as METTL3 or ALKBH5 inhibitors, has demonstrated potential to suppress CSC properties and reverse chemoresistance *in vitro* and *in vivo*, providing a foundation for novel CSC-directed therapeutic strategies (Cerneckis et al., 2023).

## 3 Development and evaluation of epigenetic drugs to overcome chemoresistance

As the critical role of epigenetic mechanisms in chemoresistance becomes increasingly evident, drug development targeting these mechanisms has emerged as a key strategy to overcome resistance. Currently, the main classes of epigenetic drugs include DNMTi, HDACi, and other emerging epigenetic modulators, such as BET protein inhibitors and histone methyltransferase inhibitors.

### 3.1 DNA methyltransferase inhibitors

DNMTi were among the first epigenetic drugs to enter clinical application. They primarily inhibit DNA methyltransferase activity, reversing aberrant DNA methylation and restoring expression of silenced genes (Zhou et al., 2018). In resistant cell lines, DNMTi can reactivate pro-apoptotic genes or drug-target genes, thereby enhancing chemotherapeutic sensitivity (Ørskov and Grønbæk, 2017). Representative drugs include Azacitidine and Decitabine, which have demonstrated efficacy in acute myeloid leukemia (AML) and myelodysplastic syndromes (MDS) (Gozdecka et al., 2025). These agents not only regulate gene expression via demethylation but also alter chromatin structure and non-coding RNA expression, providing multi-layered intervention against resistance (Gozdecka et al., 2025). For example, MC3343, a novel non-nucleoside DNMT inhibitor, suppresses osteosarcoma cell proliferation by inducing G1/G2-M phase arrest while specifically re-expressing osteogenic genes to promote differentiation, differing from conventional nucleoside DNMT inhibitors (Cristalli et al., 2022). Moreover, MC3343 synergizes with doxorubicin and cisplatin, enhancing drug-DNA binding, increasing DNA damage, and promoting tumor cell death, representing a potential strategy for patients with poor chemotherapy response (Cristalli et al., 2022).

### 3.2 Histone deacetylase inhibitors

HDACi inhibit histone deacetylase activity, increasing histone acetylation, relaxing chromatin, and activating transcription of associated genes (Sun et al., 2018). In resistant cancer cells, HDACi can upregulate pro-apoptotic genes, suppress anti-apoptotic pathways, and modulate CSC traits, thereby enhancing chemotherapeutic sensitivity (Sun et al., 2018). Common HDACi include Vorinostat and Belinostat, which have shown potential efficacy in hematologic malignancies and some solid tumors (Sun et al., 2018). Importantly, HDACi can be combined with DNMTi or conventional chemotherapy to synergistically reverse resistance. By increasing histone acetylation, HDACi make genomic DNA more accessible to cisplatin-induced damage, enhancing DNA damage, apoptosis, and cell cycle arrest, thereby improving anticancer effects (Sarkar et al., 2020). This highlights both the intrinsic anticancer potential of HDACi and the critical role of histone acetylation in regulating drug sensitivity and resistance.

### 3.3 ASO/siRNA-mediated targeting of non-coding RNAs

Recent studies have increasingly demonstrated that ncRNAs play critical roles in cancer development and chemoresistance (Ghazimoradi et al., 2023). In particular, lncRNAs and circRNAs can promote tumor tolerance to chemotherapeutic agents by regulating drug transport, apoptotic signaling, DNA damage repair, or epigenetic modifications (Ghazimoradi et al., 2023). Strategies targeting these resistance-associated ncRNAs provide new avenues to overcome chemoresistance. Antisense oligonucleotides (ASOs) and small interfering RNAs (siRNAs) are widely used RNA-targeting tools that can specifically bind target RNAs, inducing degradation or blocking their function (Chi et al., 2017). By using ASOs or siRNAs to inhibit pro-resistance lncRNAs or circRNAs, tumor cell sensitivity to chemotherapeutic drugs can be restored (Chi et al., 2017). For example, in multiple tumor models, siRNA-mediated knockdown of specific lncRNAs significantly enhances the anticancer effects of cisplatin, paclitaxel, or imatinib (Dowdy, 2023). Similarly, circRNA knockdown has been shown to reverse resistance to doxorubicin or oxaliplatin. These approaches not only directly suppress ncRNA-mediated regulation of resistance pathways but can also indirectly affect miRNA or protein complex functions, thereby broadly improving drug sensitivity (Micheletti et al., 2017). For instance, our recent study demonstrated that a PKMYT1AR-specific ASO can selectively inhibit lncRNA-PKMYT1AR expression. When combined with cisplatin, this approach synergistically suppresses lung cancer progression and reverses chemoresistance (He et al., 2021). Overall, ASO/siRNA-mediated targeting of ncRNAs provides a highly controllable and designable intervention strategy, offering potential precision therapeutic options for clinical management of chemoresistant cancers. With ongoing optimization of delivery systems and increased target specificity, such strategies are expected to see broader application in future cancer therapies.

### 3.4 RNA epigenetic modification enzyme inhibitors

#### 3.4.1 m^6^A modulators

Recent studies have shown that inhibitors targeting m^6^A regulators exhibit significant antitumor potential across multiple cancers. METTL3, an m^6^A methyltransferase, can be inhibited by STM2457, which reduces m^6^A levels on oncogenic mRNAs in AML, promotes differentiation and apoptosis, and significantly delays tumor progression *in vivo* (Yankova et al., 2021). FTO, an m^6^A demethylase, can be inhibited by FB23-2, suppressing AML proliferation, promoting differentiation/apoptosis, and inhibiting tumor growth in xenograft models (Huang Y. et al., 2019). Inhibitors of m^6^A reader proteins also show potential efficacy: SKLB-Y13, a selective YTHDF1 inhibitor, blocks binding to target mRNAs, suppressing breast cancer proliferation and inducing apoptosis (Wu et al., 2025); YTHDF2-specific inhibitors inhibit protein translation and reverse paclitaxel resistance (Liu et al., 2025). IGF2BP2 inhibitor CWI1-2 blocks m^6^A-dependent amino acid metabolism, suppressing AML stem/initiating cell self-renewal and tumor progression (Weng et al., 2022). ALKBH5 inhibitors selectively inhibit proliferation of certain leukemia and glioblastoma cells, demonstrating cell type-specific antiproliferative activity (Lv et al., 2023). Overall, these studies systematically validate that small-molecule inhibitors targeting m^6^A writers, erasers, and readers can interfere with m^6^A-dependent RNA stability and translation, suppress cancer cell proliferation, induce differentiation, enhance apoptosis, and reverse chemoresistance, highlighting m^6^A modulators as promising precision therapy strategies.

#### 3.4.2 ac^4^C modulators

N4-acetylcytidine and its catalytic enzyme NAT10 play oncogenic roles in multiple cancers and are emerging therapeutic targets (Wang Q. et al., 2025). NAT10 promotes mRNA ac^**4**^C modification, enhancing translation of MYC, CDK2, DNMT1, and serine metabolism genes (e.g., SLC1A4, HOXA9, MENIN), sustaining leukemia stem/initiating cell self-renewal and driving leukemogenesis (Zhang S. et al., 2024). In hepatocellular carcinoma, NAT10 stabilizes DNA:RNA hybrids, promoting homologous recombination repair and tumor progression. NAT10 also affects the tumor immune microenvironment; high expression correlates with reduced immune infiltration and poor patient survival (Xu et al., 2025). Inhibition of NAT10 via small molecules (e.g., Remodelin, fludarabine) or siRNA/nanoparticles targets its metabolic and repair functions (Dalhat et al., 2021), enhancing chemotherapy or immunotherapy sensitivity, reversing PARP inhibitor resistance, and exerting antitumor effects in AML, liver, breast, and ovarian cancers, indicating ac^**4**^C modulators as promising precision therapy agents.

#### 3.4.3 m^5^C modulators

Recent studies indicate that m^**5**^C and its regulators play critical roles in cancer chemoresistance and progression (Chen X. et al., 2025). NSUN2, an m^**5**^C methyltransferase, reprograms alternative splicing of SRSF6 mRNA in anaplastic thyroid carcinoma (ATC), producing AGX2 splice variants of UAP1, enhancing N-glycosylation and stabilizing ABC transporters, promoting multidrug resistance (Hou et al., 2025). NSUN2 inhibition reduces enzyme activity and downstream gene expression, overcoming resistance (Hou et al., 2025). The m5C reader YBX1 regulates gemcitabine resistance in pancreatic ductal adenocarcinoma via the YBX1-LRP1-β-catenin-RRM1 axis; YBX1 inhibitor SU056 effectively reverses resistance and enhances chemotherapy efficacy (Li et al., 2024). NSUN6 selectively methylates specific mRNA cytosine residues to promote transcription, and its structural elucidation provides a basis for small-molecule inhibitor development, with competitive binding inhibiting enzymatic activity, offering a therapeutic strategy for NSUN6-driven cancers (Zhong et al., 2025). Overall, inhibitors of m^**5**^C writers and readers can disrupt m^**5**^C-dependent splicing, transcription, and resistance pathways, suppress cancer proliferation, and reverse chemoresistance, providing new avenues for precision anticancer therapy.

## 4 Combination strategies of epigenetic modulators and chemotherapeutic agents

With a deeper understanding of the role of epigenetic mechanisms in chemoresistance, combining epigenetic drugs with conventional chemotherapeutics has emerged as a promising strategy to overcome resistance (Kryczka et al., 2021). The core principle of combination therapy is to simultaneously target multiple resistance pathways or reverse epigenetic states associated with chemoresistance, thereby enhancing drug sensitivity and improving therapeutic efficacy.

### 4.1 Preclinical studies and combination therapy potential of HDAC inhibitors

In preclinical studies, HDACis have shown limited efficacy as monotherapy for solid tumors, yet they reveal significant antitumor potential and mechanistic insights. For instance, Romidepsin induces apoptosis and G2/M cell cycle arrest in hepatocellular carcinoma via modulation of the JNK/c-Jun/caspase-3 and Erk/cdc25C/cdc2/cyclinB pathways (Sun et al., 2017); Belinostat exerts anticancer effects in KRAS-mutant lung cancer through regulation of the tricarboxylic acid cycle, urea cycle, and NRF2 signaling (Peter et al., 2023); in basal-like breast cancer (Pradyuth et al., 2023), HDACis promote KLF5 ubiquitination and degradation, demonstrating molecular subtype-specific regulatory capacity (Kong et al., 2022). While these findings have not directly translated into clinical practice, they provide theoretical support for combination therapy strategies. HDACis can potentiate the efficacy of chemotherapeutic agents primarily by remodeling chromatin structure, thereby enhancing DNA damage-induced cytotoxicity (Grumetti et al., 2022). For example, in combination with cisplatin, HDACis facilitate chromatin relaxation, promote platinum–DNA adduct formation, and suppress Bcl-2/XIAP expression; when combined with doxorubicin, they increase the number of binding sites, augmenting overall cytotoxicity (Asgar et al., 2016). HDACis also enhance targeted therapy efficacy, showing synergistic antitumor activity with BET inhibitors, topoisomerase inhibitors, IKK inhibitors, and receptor tyrosine kinase pathway inhibitors across various solid tumor models, including glioblastoma, indicating potential clinical translatability (Gusyatiner et al., 2021; Marchion et al., 2005; Dai et al., 2011; Zhao et al., 2022). Overall, HDACis exhibit potent sensitization effects in chemotherapy, exerting multi-pathway, multi-targeted antitumor effects and highlighting their multidimensional therapeutic potential in solid tumors.

### 4.2 Clinical trials of DNMTi or HDACi combinations in solid tumors

Despite their demonstrated antitumor potential in preclinical studies, HDAC inhibitors (HDACis) have shown limited efficacy as monotherapy for solid tumors in clinical settings (To et al., 2025). Preclinical models often fail to fully replicate clinical conditions due to small sample sizes, standardized experimental settings, and differences in tolerability between animals and humans; therefore, preclinical results should be interpreted as reference points requiring clinical validation (Lanzi and Cassinelli, 2022). Recently, several selective HDACis have entered clinical trials, showing promising results, particularly in HR+/HER2-breast cancer (Wang T. et al., 2023). Tucidinostat or Entinostat in combination with the aromatase inhibitor Exemestane significantly prolongs progression-free survival (PFS), with early-phase trials reporting disease control rates (DCR) of 100% and overall response rates (ORR) up to 75%, and has received Class I recommendation in Chinese clinical guidelines (Sabnis et al., 2013; Connolly et al., 2021; Yardley et al., 2013). However, in certain triple-negative breast cancer (TNBC) and multi-drug combination regimens, clinical benefit has been limited and toxicity increased, highlighting the need for optimized, individualized combination strategies (Maccallini et al., 2022; Li et al., 2021). Myelodysplastic/myeloproliferative neoplasms (MDS/MPN) are rare hematologic malignancies with poor outcomes, and DNA methyltransferase inhibitors (DNMTis) remain the only approved disease-modifying therapy for dysplastic CMML. The ABNL-MARRO 001 Phase I/II trial is evaluating the JAK1 inhibitor itacitinib combined with ASTX727 (oral decitabine/cedazuridine) to assess safety, efficacy, and biomarkers of response (Moyo et al., 2022). In solid tumors, HDACi combinations have shown promise: the Phase II CAPability-01 trial demonstrated that sintilimab plus chidamide and bevacizumab improved 18-week progression-free survival (64% vs. 21.7%) and overall response rate (44% vs. 13%) in MSS/pMMR colorectal cancer (NCT04724239) (Wang F. et al., 2024). However, in hematologic malignancies, entinostat combined with pembrolizumab showed limited efficacy and substantial toxicity in advanced MDS/AML (NCT02936752), likely due to insufficient modulation of myeloid-derived suppressor cells (Bewersdorf et al., 2024). These findings highlight the potential of DNMTi and HDACi combinations, while emphasizing the need for rational design, predictive biomarkers, and careful toxicity management.

### 4.3 Challenges of combining HDAC inhibitors with chemotherapy

While HDACis exhibit antitumor activity in certain solid tumors, their clinical application is constrained by multiple factors, including toxicity, tumor heterogeneity, and off-target effects (Schelker et al., 2023). Although generally manageable, toxicities—such as cardiotoxicity, hematologic, gastrointestinal, hepatic, and pulmonary adverse effects—may limit dosing; combination therapies can also result in severe adverse events, emphasizing the need for careful monitoring and the development of predictive biomarkers (Xu et al., 2024; Bayat et al., 2018; Parveen et al., 2023). Tumor heterogeneity leads to differential HDAC expression, influencing drug efficacy and potentially diminishing the effectiveness of combination with immunotherapy or single-target agents (Huang M. et al., 2019). Off-target effects may activate pro-oncogenic signaling pathways, necessitating the incorporation of complementary targeted strategies (Shi et al., 2024). Future development of HDACis should focus on four directions: (i) optimizing combination therapies to enhance synergistic effects and overcome resistance; (ii) developing novel drug delivery systems or formulations to improve tumor-targeted delivery and bioavailability; (iii) designing selective or isoform-specific HDACis to increase target specificity and reduce adverse effects; and (iv) identifying precise predictive biomarkers to enable individualized therapy. These strategies aim to maximize efficacy while ensuring safety, ultimately promoting the integration of HDACis into precision oncology approaches for solid tumors.

## 5 Conclusion and future perspectives

Epigenetic alterations play central roles in chemoresistance by regulating gene expression, signaling pathways, and CSC properties, thereby significantly affecting tumor drug sensitivity. Epigenetic drugs targeting these mechanisms, including DNMTi, HDACi, and emerging BET protein or RNA-modifying enzyme inhibitors, have shown potential to reverse resistance and enhance chemotherapy efficacy in preclinical studies and early clinical trials (Suraweera et al., 2025; Sun et al., 2023). Notably, combination strategies with conventional chemotherapeutics can intervene at multiple resistance levels, providing new avenues to overcome tumor chemoresistance.

Emerging evidence suggests that several molecular biomarkers may serve as promising predictors of therapeutic response to DNMTis and m^6^A-modifying enzyme inhibitors. For DNMTis, DNA methylation signatures at specific gene promoters, such as p16^INK4A^, MGMT, and MLH1, have been associated with heightened drug sensitivity, as hypermethylated loci can be reactivated upon treatment, restoring tumor suppressor functions and enhancing apoptosis (Wettergren et al., 2025; Sanchez et al., 2017). Additionally, global hypomethylation levels and specific CpG island methylator phenotypes (CIMP) have been correlated with improved clinical response in hematologic malignancies and selected solid tumors (Teodoridis et al., 2008; Park et al., 2024). In the context of m^6^A inhibitors, the expression levels and activity of m^6^A “writers” (e.g., METTL3, METTL14), “erasers” (e.g., FTO, ALKBH5), and “readers” (e.g., YTHDF1-2) appear to influence therapeutic outcomes. High METTL3 expression, for example, has been linked to increased dependency of tumor cells on m^6^A-mediated RNA regulation, rendering them more susceptible to m^6^A inhibition (Wang J. et al., 2024; Fang M. et al., 2025). Furthermore, integrative profiling of downstream m^6^A-modified transcripts, such as oncogenes or regulators of apoptosis and immune evasion, may refine patient stratification and predict responsiveness more accurately (Su et al., 2020; Chen Z. et al., 2025; Lin et al., 2023). Collectively, these biomarkers highlight a precision-medicine approach in the use of epigenetic and epitranscriptomic inhibitors, offering a framework for rational patient selection and optimized clinical outcomes.

Future research should focus on: (i) development of precise biomarkers to predict patient response and enable personalized epigenetic therapy; (ii) optimization of combination regimens, exploring synergistic mechanisms and dosing strategies to maximize efficacy while minimizing toxicity; and (iii) conducting systematic clinical trials to evaluate the efficacy and safety of epigenetic drugs and combination therapies across different cancer types and resistance profiles. Through these efforts, epigenetic interventions are poised to become integral to future strategies for treating chemoresistant cancers, offering new opportunities to improve patient survival and prognosis.

## References

[B1] Al-Imam M. J. Hussein U. A. Sead F. F. Faqri A. M. A. Mekkey S. M. Khazel A. J. (2023). The interactions between DNA methylation machinery and long non-coding RNAs in tumor progression and drug resistance. DNA Repair 128, 103526. 10.1016/j.dnarep.2023.103526 37406581

[B2] Armache A. Yang S. Martínez de Paz A. Robbins L. E. Durmaz C. Cheong J. Q. (2020). Histone H3.3 phosphorylation amplifies stimulation-induced transcription. Nature 583, 852–857. 10.1038/s41586-020-2533-0 32699416 PMC7517595

[B3] Asgar M. A. Senawong G. Sripa B. Senawong T. (2016). Synergistic anticancer effects of cisplatin and histone deacetylase inhibitors (SAHA and TSA) on cholangiocarcinoma cell lines. Int. Journal Oncology 48, 409–420. 10.3892/ijo.2015.3240 26575528

[B4] Bayat S. Shekari Khaniani M. Choupani J. Alivand M. R. Mansoori Derakhshan S. (2018). HDACis (class I), cancer stem cell, and phytochemicals: cancer therapy and prevention implications. Biomed. and Pharmacotherapy = Biomedecine and Pharmacotherapie 97, 1445–1453. 10.1016/j.biopha.2017.11.065 29156535

[B5] Bewersdorf J. P. Shallis R. M. Sharon E. Park S. Ramaswamy R. Roe C. E. (2024). A multicenter phase Ib trial of the histone deacetylase inhibitor entinostat in combination with pembrolizumab in patients with myelodysplastic syndromes/neoplasms or acute myeloid leukemia refractory to hypomethylating agents. Ann. Hematology 103, 105–116. 10.1007/s00277-023-05552-4 38036712 PMC11838822

[B6] Bilmez Y. Talibova G. Ozturk S. (2022). Dynamic changes of histone methylation in Mammalian oocytes and early embryos. Histochem. Cell Biology 157, 7–25. 10.1007/s00418-021-02036-2 34599660

[B7] Cerneckis J. Cui Q. Liu W. Shi Y. (2023). RNA modifications in cancer stem cell biology. Cancer Treatment Research 190, 25–47. 10.1007/978-3-031-45654-1_2 38112998

[B8] Chen D. Gu X. Nurzat Y. Xu L. Li X. Wu L. (2024). Writers, readers, and erasers RNA modifications and drug resistance in cancer. Mol. Cancer 23, 178. 10.1186/s12943-024-02089-6 39215288 PMC11363509

[B9] Chen X. Yuan Y. Zhou F. Huang X. Li L. Pu J. (2025a). RNA m5C modification: from physiology to pathology and its biological significance. Front. Immunology 16, 1599305. 10.3389/fimmu.2025.1599305 40370440 PMC12075115

[B10] Chen Z. Zeng C. Yang L. Che Y. Chen M. Sau L. (2025b). YTHDF2 promotes ATP synthesis and immune evasion in B cell malignancies. Cell 188, 331–351.e30. 10.1016/j.cell.2024.11.007 39694037 PMC12394000

[B11] Chi X. Gatti P. Papoian T. (2017). Safety of antisense oligonucleotide and siRNA-based therapeutics. Drug Discovery Today 22, 823–833. 10.1016/j.drudis.2017.01.013 28159625

[B12] Christmann M. Kaina B. (2019). Epigenetic regulation of DNA repair genes and implications for tumor therapy. Mutation research. Rev. Mutation Research 780, 15–28. 10.1016/j.mrrev.2017.10.001 31395346

[B13] Connolly R. M. Zhao F. Miller K. D. Lee M. J. Piekarz R. L. Smith K. L. (2021). E2112: randomized phase III trial of endocrine therapy plus entinostat or placebo in hormone receptor-positive advanced breast cancer. A trial of the ECOG-ACRIN cancer research group. J. Clinical Oncology Official Journal Am. Soc. Clin. Oncol. 39, 3171–3181. 10.1200/JCO.21.00944 34357781 PMC8478386

[B14] Cristalli C. Manara M. C. Valente S. Pellegrini E. Bavelloni A. De Feo A. (2022). Novel targeting of DNA methyltransferase activity inhibits ewing sarcoma cell proliferation and enhances tumor cell sensitivity to DNA damaging drugs by activating the DNA damage response. Front. Endocrinology 13, 876602. 10.3389/fendo.2022.876602 35712255 PMC9197596

[B15] Dai Y. Chen S. Wang L. Pei X. Y. Funk V. L. Kramer L. B. (2011). Disruption of IkappaB kinase (IKK)-Mediated RelA serine 536 phosphorylation sensitizes human multiple myeloma cells to histone deacetylase (HDAC) inhibitors. J. Biological Chemistry 286, 34036–34050. 10.1074/jbc.M111.284216 21816815 PMC3190767

[B16] Dalhat M. H. Altayb H. N. Khan M. I. Choudhry H. (2021). Structural insights of human N-acetyltransferase 10 and identification of its potential novel inhibitors. Sci. Reports 11, 6051. 10.1038/s41598-021-84908-0 33723305 PMC7960695

[B17] Delaunay S. Helm M. Frye M. (2024). RNA modifications in physiology and disease: towards clinical applications. Nat. Reviews. Genet. 25, 104–122. 10.1038/s41576-023-00645-2 37714958

[B18] Dixit D. Prager B. C. Gimple R. C. Poh H. X. Wang Y. Wu Q. (2021). The RNA m6A reader YTHDF2 maintains oncogene expression and is a targetable dependency in glioblastoma stem cells. Cancer Discovery 11, 480–499. 10.1158/2159-8290.CD-20-0331 33023892 PMC8110214

[B19] Dowdy S. F. (2023). Endosomal escape of RNA therapeutics: how do we solve this rate-limiting problem? RNA (New York, N.Y.) 29, 396–401. 10.1261/rna.079507.122 36669888 PMC10019367

[B20] Fang X. Cai Y. Peng X. Li Z. Huang M. Li Y. (2025a). Epicatechin attenuates the stemness of liver cancer stem cells and tumorigenesis through DNA methylation-mediated inactivation of GINS1/HRAS. J. Translational Medicine 23, 828. 10.1186/s12967-025-06790-y 40713555 PMC12291481

[B21] Fang M. Li Y. Wang P. Wang Y. Wang X. Wa X. (2025b). METTL3 inhibition restores PD-L1 expression and CD8+ T-cell cytotoxic function in immunotherapy-treated gastric cancer. Cancer Immunology Research 13, 1037–1052. 10.1158/2326-6066.CIR-24-1179 40299705

[B22] Fontecha-Barriuso M. Martin-Sanchez D. Ruiz-Andres O. Poveda J. Sanchez-Niño M. D. Valiño-Rivas L. (2018). Targeting epigenetic DNA and histone modifications to treat kidney disease. Nephrol. Dialysis, Transplantation 33, 1875–1886. 10.1093/ndt/gfy009 29534238

[B23] Gao K. Li X. Ni J. Wu B. Guo J. Zhang R. (2023). Non-coding RNAs in enzalutamide resistance of castration-resistant prostate cancer. Cancer Letters 566, 216247. 10.1016/j.canlet.2023.216247 37263338

[B24] Ghazimoradi M. H. Karimpour-Fard N. Babashah S. (2023). The promising role of non-coding RNAs as biomarkers and therapeutic targets for leukemia. Genes 14, 131. 10.3390/genes14010131 36672872 PMC9859176

[B25] Gozdecka M. Dudek M. Wen S. Gu M. Stopforth R. J. Rak J. (2025). Mitochondrial metabolism sustains DNMT3A-R882-mutant clonal haematopoiesis. Nature 642, 431–441. 10.1038/s41586-025-08980-6 40239706 PMC12158785

[B26] Grumetti L. Lombardi R. Iannelli F. Pucci B. Avallone A. Di Gennaro E. (2022). Epigenetic approaches to overcome fluoropyrimidines resistance in solid tumors. Cancers 14, 695. 10.3390/cancers14030695 35158962 PMC8833539

[B27] Gu Y. Yang R. Zhang Y. Guo M. Takehiro K. Zhan M. (2025). Molecular mechanisms and therapeutic strategies in overcoming chemotherapy resistance in cancer. Mol. Biomedicine 6, 2. 10.1186/s43556-024-00239-2 39757310 PMC11700966

[B28] Gusyatiner O. Bady P. Pham M. D. T. Lei Y. Park J. Daniel R. T. (2021). BET inhibitors repress expression of interferon-stimulated genes and synergize with HDAC inhibitors in glioblastoma. Neuro-oncology 23, 1680–1692. 10.1093/neuonc/noab115 33987681 PMC8485441

[B29] He Y. Jiang X. Duan L. Xiong Q. Yuan Y. Liu P. (2021). LncRNA PKMYT1AR promotes cancer stem cell maintenance in non-small cell lung cancer *via* activating wnt signaling pathway. Mol. Cancer 20, 156. 10.1186/s12943-021-01469-6 34856993 PMC8638142

[B30] Hombach S. Kretz M. (2016). Non-coding RNAs: classification, biology and functioning. Adv. Experimental Medicine Biology 937, 3–17. 10.1007/978-3-319-42059-2_1 27573892

[B31] Hou X. Dong Q. Hao J. Liu M. Ning J. Tao M. (2025). NSUN2-mediated m(5)C modification drives alternative splicing reprogramming and promotes multidrug resistance in Anaplastic thyroid cancer through the NSUN2/SRSF6/UAP1 signaling axis. Theranostics 15, 2757–2777. 10.7150/thno.104713 40083919 PMC11898302

[B32] Hu C. Liu X. Zeng Y. Liu J. Wu F. (2021). DNA methyltransferase inhibitors combination therapy for the treatment of solid tumor: mechanism and clinical application. Clin. Epigenetics 13, 166. 10.1186/s13148-021-01154-x 34452630 PMC8394595

[B33] Huang Y. Su R. Sheng Y. Dong L. Dong Z. Xu H. (2019a). Small-molecule targeting of oncogenic FTO demethylase in acute myeloid leukemia. Cancer Cell 35, 677–691.e10. 10.1016/j.ccell.2019.03.006 30991027 PMC6812656

[B34] Huang M. Zhang J. Yan C. Li X. Zhang J. Ling R. (2019b). Small molecule HDAC inhibitors: promising agents for breast cancer treatment. Bioorg. Chemistry 91, 103184. 10.1016/j.bioorg.2019.103184 31408831

[B35] Ji H. Elangbam M. Qiu Y. Bamrah J. Zhang W. Pawar A. (2025). Arsenic disrupts H3K9me3 and H3K27me3 balance by biasing PRC2.1 and PRC2.2 activity *via* PALI1 inhibition in carcinogenesis. Int. Journal Biological Sciences 21, 4069–4080. 10.7150/ijbs.115605 40612682 PMC12223765

[B36] Jiang X. Liu B. Nie Z. Duan L. Xiong Q. Jin Z. (2021). The role of m6A modification in the biological functions and diseases. Signal Transduction Targeted Therapy 6, 74. 10.1038/s41392-020-00450-x 33611339 PMC7897327

[B37] Jin M. L. Jeong K. W. (2023). Histone modifications in drug-resistant cancers: from a cancer stem cell and immune evasion perspective. Exp. and Molecular Medicine 55, 1333–1347. 10.1038/s12276-023-01014-z 37394580 PMC10394043

[B38] Kong Y. Ren W. Fang H. Shah N. A. Shi Y. You D. (2022). Histone deacetylase inhibitors (HDACi) promote KLF5 ubiquitination and degradation in basal-like breast cancer. Int. Journal Biological Sciences 18, 2104–2115. 10.7150/ijbs.65322 35342356 PMC8935240

[B39] Kris M. G. Hellmann M. D. Chaft J. E. (2014). “Chemotherapy for lung cancers: here to stay. American society of clinical oncology educational book,” in Annual meeting. American Society of Clinical Oncology, e375–e380. 10.14694/EdBook_AM.2014.34.e375 24857127

[B40] Kryczka J. Kryczka J. Czarnecka-Chrebelska K. H. Brzeziańska-Lasota E. (2021). Molecular mechanisms of chemoresistance induced by cisplatin in NSCLC cancer therapy. Int. Journal Molecular Sciences 22, 8885. 10.3390/ijms22168885 34445588 PMC8396273

[B41] Kulis M. Esteller M. (2010). DNA methylation and cancer. Adv. Genetics 70, 27–56. 10.1016/B978-0-12-380866-0.60002-2 20920744

[B42] Li F. Si W. Xia L. Yin D. Wei T. Tao M. (2024). Positive feedback regulation between glycolysis and histone lactylation drives oncogenesis in pancreatic ductal adenocarcinoma. Mol. Cancer 23, 90. 10.1186/s12943-024-02008-9 38711083 PMC11071201

[B43] Lanzi C. Cassinelli G. (2022). Combinatorial strategies to potentiate the efficacy of HDAC inhibitors in fusion-positive sarcomas. Biochem. Pharmacology 198, 114944. 10.1016/j.bcp.2022.114944 35152144

[B44] Li M. Viswanadhapalli S. Santhamma B. Pratap U. P. Luo Y. Liu J. (2021). LIFR inhibition enhances the therapeutic efficacy of HDAC inhibitors in triple negative breast cancer. Commun. Biology 4, 1235. 10.1038/s42003-021-02741-7 34716410 PMC8556368

[B45] Li B. Xing F. Wang J. Wang X. Zhou C. Fan G. (2024). YBX1 as a therapeutic target to suppress the LRP1-β-catenin-RRM1 axis and overcome gemcitabine resistance in pancreatic cancer. Cancer Letters 602, 217197. 10.1016/j.canlet.2024.217197 39216548

[B46] Lin W. Chen L. Zhang H. Qiu X. Huang Q. Wan F. (2023). Tumor-intrinsic YTHDF1 drives immune evasion and resistance to immune checkpoint inhibitors *via* promoting MHC-I degradation. Nat. Communications 14, 265. 10.1038/s41467-022-35710-7 36650153 PMC9845301

[B47] Liu T. Yang D. Wei Q. Wang Y. Tian L. Liu X. (2025). The RNA-stability-independent role of the RNA m(6)A reader YTHDF2 in promoting protein translation to confer tumor chemotherapy resistance. Mol. Cell 85, 2320–2336.e9. 10.1016/j.molcel.2025.05.015 40480228

[B48] Luo F. Zhang M. Sun B. Xu C. Yang Y. Zhang Y. (2024). LINC00115 promotes chemoresistant breast cancer stem-like cell stemness and metastasis through SETDB1/PLK3/HIF1α signaling. Mol. Cancer 23, 60. 10.1186/s12943-024-01975-3 38520019 PMC10958889

[B49] Lv D. Zhong C. Dixit D. Yang K. Wu Q. Godugu B. (2023). EGFR promotes ALKBH5 nuclear retention to attenuate N6-methyladenosine and protect against ferroptosis in glioblastoma. Mol. Cell 83, 4334–4351.e7. 10.1016/j.molcel.2023.10.025 37979586 PMC10842222

[B50] Maccallini C. Ammazzalorso A. De Filippis B. Fantacuzzi M. Giampietro L. Amoroso R. (2022). HDAC inhibitors for the therapy of triple negative breast cancer. Pharm. Basel, Switz. 15, 667. 10.3390/ph15060667 35745586 PMC9230362

[B51] Marchion D. C. Bicaku E. Turner J. G. Daud A. I. Sullivan D. M. Munster P. N. (2005). Synergistic interaction between histone deacetylase and topoisomerase II inhibitors is mediated through topoisomerase IIbeta. Clin. Cancer Research An Official Journal Am. Assoc. Cancer Res. 11, 8467–8475. 10.1158/1078-0432.CCR-05-1073 16322310

[B52] Mattiroli F. Penengo L. (2021). Histone ubiquitination: an integrative signaling platform in genome stability. Trends Genetics TIG 37, 566–581. 10.1016/j.tig.2020.12.005 33485674

[B53] Micheletti R. Plaisance I. Abraham B. J. Sarre A. Ting C. C. Alexanian M. (2017). The long noncoding RNA wisper controls cardiac fibrosis and remodeling. Sci. Translational Medicine 9 (395). 10.1126/scitranslmed.aai9118 28637928 PMC5643582

[B54] Milan T. M. Eskenazi A. P. E. Bighetti-Trevisan R. L. de Almeida L. O. (2022). Epigenetic modifications control loss of adhesion and aggressiveness of cancer stem cells derived from head and neck squamous cell carcinoma with intrinsic resistance to cisplatin. Archives Oral Biology 141, 105468. 10.1016/j.archoralbio.2022.105468 35679799

[B55] Moore L. D. Le T. Fan G. (2013). DNA methylation and its basic function. Neuropsychopharmacol. Official Publication Am. Coll. Neuropsychopharmacol. 38, 23–38. 10.1038/npp.2012.112 22781841 PMC3521964

[B56] Mosca L. Ilari A. Fazi F. Assaraf Y. G. Colotti G. (2021). Taxanes in cancer treatment: activity, chemoresistance and its overcoming. Drug Resistance Updates Reviews Commentaries Antimicrobial Anticancer Chemotherapy 54, 100742. 10.1016/j.drup.2020.100742 33429249

[B57] Moyo T. K. Mendler J. H. Itzykson R. Kishtagari A. Solary E. Seegmiller A. C. (2022). The ABNL-MARRO 001 study: a phase 1-2 study of randomly allocated active myeloid target compound combinations in MDS/MPN overlap syndromes. BMC Cancer 22, 1013. 10.1186/s12885-022-10073-w 36153475 PMC9509596

[B58] Narita T. Weinert B. T. Choudhary C. (2019). Functions and mechanisms of non-histone protein acetylation. Nat. Reviews. Mol. Cell Biology 20, 156–174. 10.1038/s41580-018-0081-3 30467427

[B59] Ørskov A. D. Grønbæk K. (2017). DNA methyltransferase inhibitors in myeloid cancer: clonal eradication or clonal differentiation? Cancer Journal (Sudbury, Mass.) 23, 277–285. 10.1097/PPO.0000000000000282 28926428

[B60] Park P. H. Keith K. Calendo G. Jelinek J. Madzo J. Gharaibeh R. Z. (2024). Association between gut microbiota and CpG island methylator phenotype in colorectal cancer. Gut Microbes 16, 2363012. 10.1080/19490976.2024.2363012 38860458 PMC11174071

[B61] Parveen R. Harihar D. Chatterji B. P. (2023). Recent histone deacetylase inhibitors in cancer therapy. Cancer 129, 3372–3380. 10.1002/cncr.34974 37560925

[B62] Peter R. M. Sarwar M. S. Mostafa S. Z. Wang Y. Su X. Kong A. N. (2023). Histone deacetylase inhibitor belinostat regulates metabolic reprogramming in killing KRAS-Mutant human lung cancer cells. Mol. Carcinogenesis 62, 1136–1146. 10.1002/mc.23551 37144836 PMC10524423

[B63] Pradyuth K. S. Salunkhe S. A. Singh A. K. Chitkara D. Mittal A. (2023). Belinostat loaded lipid-polymer hybrid nanoparticulate delivery system for breast cancer: improved pharmacokinetics and biodistribution in a tumor model. J. Materials Chemistry 11, 10859–10872. 10.1039/d3tb01317k 37938124

[B64] Sabnis G. J. Goloubeva O. G. Kazi A. A. Shah P. Brodie A. H. (2013). HDAC inhibitor entinostat restores responsiveness of letrozole-resistant MCF-7Ca xenografts to aromatase inhibitors through modulation of Her-2. Mol. Cancer Therapeutics 12, 2804–2816. 10.1158/1535-7163.MCT-13-0345 24092810 PMC3858401

[B65] Sanchez H. Hossain M. B. Lera L. Hirsch S. Albala C. Uauy R. (2017). High levels of circulating folate concentrations are associated with DNA methylation of tumor suppressor and repair genes p16, MLH1, and MGMT in elderly chileans. Clin. Epigenetics 9, 74. 10.1186/s13148-017-0374-y 28748002 PMC5525256

[B66] Sarkar R. Banerjee S. Amin S. A. Adhikari N. Jha T. (2020). Histone deacetylase 3 (HDAC3) inhibitors as anticancer agents: a review. Eur. Journal Medicinal Chemistry 192, 112171. 10.1016/j.ejmech.2020.112171 32163814

[B67] Sawant M. Wilson A. Sridaran D. Mahajan K. O'Conor C. J. Hagemann I. S. (2023). Epigenetic reprogramming of cell cycle genes by ACK1 promotes breast cancer resistance to CDK4/6 inhibitor. Oncogene 42, 2263–2277. 10.1038/s41388-023-02747-x 37330596 PMC10348910

[B68] Schelker C. Nowak-Sliwinska P. Borchard G. (2023). HDACIs and TKIs combinations and their liposomal delivery for cancer treatment. J. Controlled Release Official Journal Control. Release Soc. 358, 59–77. 10.1016/j.jconrel.2023.04.006 37037270

[B69] Shi M. Q. Xu Y. Fu X. Pan D. S. Lu X. P. Xiao Y. (2024). Advances in targeting histone deacetylase for treatment of solid tumors. J. Hematology and Oncology 17, 37. 10.1186/s13045-024-01551-8 38822399 PMC11143662

[B70] Shvedunova M. Akhtar A. (2022). Modulation of cellular processes by histone and non-histone protein acetylation. Nat. Reviews. Mol. Cell Biology 23, 329–349. 10.1038/s41580-021-00441-y 35042977

[B71] Sritharan S. Sivalingam N. (2025). Epigenetic modulation of doxorubicin resistance and strategies for enhancing chemotherapeutic sensitivity. Int. Review Cell Molecular Biology 390, 186–198. 10.1016/bs.ircmb.2024.09.004 39864895

[B72] Su R. Dong L. Li Y. Gao M. Han L. Wunderlich M. (2020). Targeting FTO suppresses cancer stem cell maintenance and immune evasion. Cancer Cell 38, 79–96.e11. 10.1016/j.ccell.2020.04.017 32531268 PMC7363590

[B73] Sun W. J. Huang H. He B. Hu D. H. Li P. H. Yu Y. J. (2017). Romidepsin induces G2/M phase arrest *via* Erk/cdc25C/cdc2/cyclinB pathway and apoptosis induction through JNK/c-Jun/caspase3 pathway in hepatocellular carcinoma cells. Biochem. Pharmacology 127, 90–100. 10.1016/j.bcp.2016.12.008 28012958

[B74] Sun Y. Sun Y. Yue S. Wang Y. Lu F. (2018). Histone deacetylase inhibitors in cancer therapy. Curr. Topics Medicinal Chemistry 18, 2420–2428. 10.2174/1568026619666181210152115 30526462

[B75] Sun Y. Shen W. Hu S. Lyu Q. Wang Q. Wei T. (2023). METTL3 promotes chemoresistance in small cell lung cancer by inducing mitophagy. J. Experimental and Clinical Cancer Research CR 42, 65. 10.1186/s13046-023-02638-9 36932427 PMC10022264

[B76] Suraweera A. O'Byrne K. J. Richard D. J. (2025). Epigenetic drugs in cancer therapy. Cancer Metastasis Reviews 44, 37. 10.1007/s10555-025-10253-7 40011240 PMC11865116

[B77] Teodoridis J. M. Hardie C. Brown R. (2008). CpG island methylator phenotype (CIMP) in cancer: causes and implications. Cancer Letters 268, 177–186. 10.1016/j.canlet.2008.03.022 18471961

[B78] To K. K. W. Tolu S. S. Wang L. Zhang H. Cho W. C. Bates S. E. (2025). HDAC inhibitors: cardiotoxicity and paradoxical cardioprotective effect in ischemia-reperfusion myocardiocyte injury. Seminars Cancer Biology 113, 25–38. 10.1016/j.semcancer.2025.05.008 40360097

[B79] Wainwright E. N. Scaffidi P. (2017). Epigenetics and cancer stem cells: unleashing, hijacking, and restricting cellular plasticity. Trends Cancer 3, 372–386. 10.1016/j.trecan.2017.04.004 28718414 PMC5506260

[B80] Wang A. Huang H. Shi J. H. Yu X. Ding R. Zhang Y. (2023a). USP47 inhibits m6A-dependent c-Myc translation to maintain regulatory T cell metabolic and functional homeostasis. J. Clinical Investigation 133, e169365. 10.1172/JCI169365 37788092 PMC10688989

[B81] Wang T. Shen G. Li J. Huo X. Wang M. Liu Z. (2023b). Second-line endocrine therapy of hormone Receptor-positive/HER2-negative advanced breast cancer: a systematic review and network meta-analysis. Curr. Cancer Drug Targets 23, 718–730. 10.2174/1568009623666230407101128 37026492

[B82] Wang F. Jin Y. Wang M. Luo H. Y. Fang W. J. Wang Y. N. (2024a). Combined anti-PD-1, HDAC inhibitor and anti-VEGF for MSS/pMMR colorectal cancer: a randomized phase 2 trial. Nat. Medicine 30, 1035–1043. 10.1038/s41591-024-02813-1 38438735

[B83] Wang J. Fan P. Shen P. Fan C. Zhao P. Yao S. (2024b). XBP1s activates METTL3/METTL14 for ER-phagy and paclitaxel sensitivity regulation in breast cancer. Cancer Letters 596, 216846. 10.1016/j.canlet.2024.216846 38582397

[B84] Wang Y. Frederick J. Medina K. I. Bartom E. T. Almassalha L. M. Zhang Y. (2025a). Chromatin organization governs transcriptional response and plasticity of cancer stem cells. Adv. Science Weinheim, Baden-Wurttemberg, Ger. 12, e2407426. 10.1002/advs.202407426 40051293 PMC12061297

[B85] Wang Q. Yuan Y. Zhou Q. Jia Y. Liu J. Xiao G. (2025b). RNA N4-acetylcytidine modification and its role in health and diseases. MedComm 6, e70015. 10.1002/mco2.70015 39764566 PMC11702397

[B86] Weng H. Huang F. Yu Z. Chen Z. Prince E. Kang Y. (2022). The m(6)A reader IGF2BP2 regulates glutamine metabolism and represents a therapeutic target in acute myeloid leukemia. Cancer Cell 40, 1566–1582.e10. 10.1016/j.ccell.2022.10.004 36306790 PMC9772162

[B87] Wettergren Y. Rolny P. Lindegren H. Odin E. Rotter Sopasakis V. Keane S. (2025). Increased MLH1, MGMT, and p16INK4a methylation levels in Colon mucosa potentially useful as early risk marker of Colon cancer. Mol. and Cellular Oncology 12, 2503069. 10.1080/23723556.2025.2503069 40357388 PMC12068326

[B88] Wu Y. Feng G. Shuai W. Yang X. Pan X. Zhu C. (2025). Identification of a selective YTHDF1 inhibitor targeting the m(6)A recognition domain for breast cancer. Angewandte Chemie Int. Ed. Engl. 64, e202509316. 10.1002/anie.202509316 40827557

[B89] Xu T. Fang Y. Gu Y. Xu D. Hu T. Yu T. (2024). HDAC inhibitor SAHA enhances antitumor immunity *via* the HDAC1/JAK1/FGL1 axis in lung adenocarcinoma. J. Immunotherapy Cancer 12, e010077. 10.1136/jitc-2024-010077 39384195 PMC11474878

[B90] Xu Z. Zhu M. Geng L. Zhang J. Xia J. Wang Q. (2025). Targeting NAT10 attenuates homologous recombination *via* destabilizing DNA:RNA hybrids and overcomes PARP inhibitor resistance in cancers. Drug Resistance Updates Reviews Commentaries Antimicrobial Anticancer Chemotherapy 81, 101241. 10.1016/j.drup.2025.101241 40132530

[B91] Yankova E. Blackaby W. Albertella M. Rak J. De Braekeleer E. Tsagkogeorga G. (2021). Small-molecule inhibition of METTL3 as a strategy against myeloid leukaemia. Nature 593, 597–601. 10.1038/s41586-021-03536-w 33902106 PMC7613134

[B92] Yardley D. A. Ismail-Khan R. R. Melichar B. Lichinitser M. Munster P. N. Klein P. M. (2013). Randomized phase II, double-blind, placebo-controlled study of exemestane with or without entinostat in postmenopausal women with locally recurrent or metastatic estrogen receptor-positive breast cancer progressing on treatment with a nonsteroidal aromatase inhibitor. J. Clinical Oncology Official Journal Am. Soc. Clin. Oncol. 31, 2128–2135. 10.1200/JCO.2012.43.7251 23650416 PMC4881332

[B93] Yeldag G. Rice A. Del Río Hernández A. (2018). Chemoresistance and the self-maintaining tumor microenvironment. Cancers 10 (12), 471. 10.3390/cancers10120471 30487436 PMC6315745

[B94] Yuan Y. Wu Y. Li C. Huang Z. Peng D. Wu Z. (2025). Circ0515 reprogramming mitochondrial succinate metabolism and promotes lung adenocarcinoma progression through regulating SDHB. Cell Death and Disease 16, 497. 10.1038/s41419-025-07830-7 40617805 PMC12228733

[B95] Zaib S. Rana N. Khan I. (2022). Histone modifications and their role in epigenetics of cancer. Curr. Medicinal Chemistry 29, 2399–2411. 10.2174/0929867328666211108105214 34749606

[B96] Zhang X. Su T. Wu Y. Cai Y. Wang L. Liang C. (2024a). N6-Methyladenosine reader YTHDF1 promotes stemness and therapeutic resistance in hepatocellular carcinoma by enhancing NOTCH1 expression. Cancer Research 84, 827–840. 10.1158/0008-5472.CAN-23-1916 38241695

[B97] Zhang S. Huang F. Wang Y. Long Y. Li Y. Kang Y. (2024b). NAT10-mediated mRNA N(4)-acetylcytidine reprograms serine metabolism to drive leukaemogenesis and stemness in acute myeloid leukaemia. Nat. Cell Biology 26, 2168–2182. 10.1038/s41556-024-01548-y 39506072 PMC11628400

[B98] Zhao L. M. Zhang J. H. (2019). Histone deacetylase inhibitors in tumor immunotherapy. Curr. Medicinal Chemistry 26, 2990–3008. 10.2174/0929867324666170801102124 28762309

[B99] Zhao T. He L. Wong L. P. Mei S. Xia J. Xu Y. (2025). Derepressing nuclear pyruvate dehydrogenase induces therapeutic cancer cell reprogramming. Cell Metab. 37, 1667–1681.e13. 10.1016/j.cmet.2025.05.009 40505660 PMC13717923

[B100] Zhao L. Y. Song J. Liu Y. Song C. X. Yi C. (2020). Mapping the epigenetic modifications of DNA and RNA. Protein and Cell 11, 792–808. 10.1007/s13238-020-00733-7 32440736 PMC7647981

[B101] Zhao L. Liang Q. He Y. Liu M. Tong R. Jiang Z. (2022). HDAC/JAK dual target inhibitors of cancer-related targets: the success of nonclearable linked pharmacophore mode. Bioorg. Chemistry 129, 106181. 10.1016/j.bioorg.2022.106181 36302332

[B102] Zhong F. Pu T. Hu Q. Li M. Wang L. Wang S. (2025). NSUN6 inhibitor discovery guided by its mRNA substrate bound crystal structure. Struct. Lond. Engl. 33, 443–450.e4. 10.1016/j.str.2024.12.021 39862858

[B103] Zhou Z. Li H. Q. Liu F. (2018). DNA methyltransferase inhibitors and their therapeutic potential. Curr. Topics Medicinal Chemistry 18, 2448–2457. 10.2174/1568026619666181120150122 30465505

[B104] Zhou H. Qin D. Xie C. Zhou J. Jia S. Zhou Z. (2024). Combinations of HDAC inhibitor and PPAR agonist induce ferroptosis of leukemic stem cell-like cells in acute myeloid leukemia. Clin. Cancer Research An Official Journal Am. Assoc. Cancer Res. 30, 5430–5444. 10.1158/1078-0432.CCR-24-0796 39321217

